# Different electrophysiology patterns in GNE myopathy

**DOI:** 10.1186/s13023-022-02355-0

**Published:** 2022-05-19

**Authors:** Xiangyi Liu, Yingshuang Zhang, Shuo Zhang, Aping Sun, Danfeng Zheng, Dongsheng Fan, Xiaoxuan Liu

**Affiliations:** Department of Neurology, Peking University Third Hospital, No. 49, North Garden Road, Haidian District, Beijing, 100191 China

**Keywords:** GNE myopathy, Neurogenic pattern, Novel mutation, Muscle pathology

## Abstract

**Background:**

GNE myopathy is a rare distal myopathy caused by mutations of the *GNE* gene. A few cases of GNE myopathy accompanied by neurogenic features of electrophysiology mimicking hereditary motor neuropathy were reported recently. We confirmed this feature and described the clinical phenotype and mutations of GNE myopathy in these rare cases.

**Results:**

The absence of lower limb tendon reflexes, decreased compound muscle action potentials in lower leg motor nerves, and neurogenic pattern of electromyography suggested neuropathy in four patients. However, muscle pathology revealed a predominantly myogenic pattern. The follow-up electroneurography results implied that the compound motor action potential amplitudes deteriorated over time. Next-generation sequencing identified three novel variants of the *GNE* gene, c.2054T > C (p.Val685Ala), c.424G > A (p.Gly142Arg) and c.944T > C (p.Phe315Ser), as well as two hotspot mutations, c.115C > T(p.Arg39*) and c.620A > T(p.Asp207Val), in these patients. These novel mutations cosegregated with disease in the family.

**Conclusions:**

These rare cases supported the existence of neurogenic features of electrophysiology different from the typical myopathic pattern of GNE myopathy.

**Supplementary Information:**

The online version contains supplementary material available at 10.1186/s13023-022-02355-0.

## Background

GNE myopathy (OMIM 605820), also known as distal myopathy with rimmed vacuoles (DMRV), is a rare distal myopathy caused by mutations of the *GNE* gene in an autosomal recessive inheritance pattern [1, 2]. The highly conserved *GNE* gene encodes the bifunctional enzyme UDP-N-acetylglucosamine 2-epimerase/N-acetylmannosamine kinase, which catalyzes the first two steps of sialic acid biosynthesis [3]. More than 260 mutations in the *GNE* gene have been identified worldwide, and the majority are missense [4]. The founder effect of *GNE* mutations has been reported in the Middle Eastern (p.Met743Thr), Japanese, and Chinese (p.Asp207Val) populations, as well other ethnicities [5, 6].

Sialic acid is crucial for glycoprotein and glycolipid synthesis; however, only skeletal muscles are verified to be affected in GNE myopathy due to tissue-specific expression of sialic acid. It is possible that other tissues are involved in GNE myopathy, given that sialic acid is essential for many biological processes [7]. Thrombocytopenia and cardiomyopathy have been reported in a few GNE myopathy cases [8, 9], but questions have been raised about whether they are rare features of the disease or were observed purely by coincidence. A few cases of GNE myopathy accompanied by motor axonal neuropathy, mimicking hereditary motor neuropathy, were reported recently [10, 11]. Although neurogenic features were observed in these studies, whether motor axonal nerves are involved in GNE myopathy remains unknown.

In this study, we report a series of cases of GNE myopathy accompanied by neurogenic features of electrophysiology mimicking hereditary motor neuropathy and describe the clinical characteristics of these cases, as well as novel *GNE* mutations. We emphasize the importance of recognizing this rare phenomenon in GNE myopathy and the challenge of differentiating it from hereditary neuropathies.

## Results

### Clinical characteristics

**Fig. 1:**
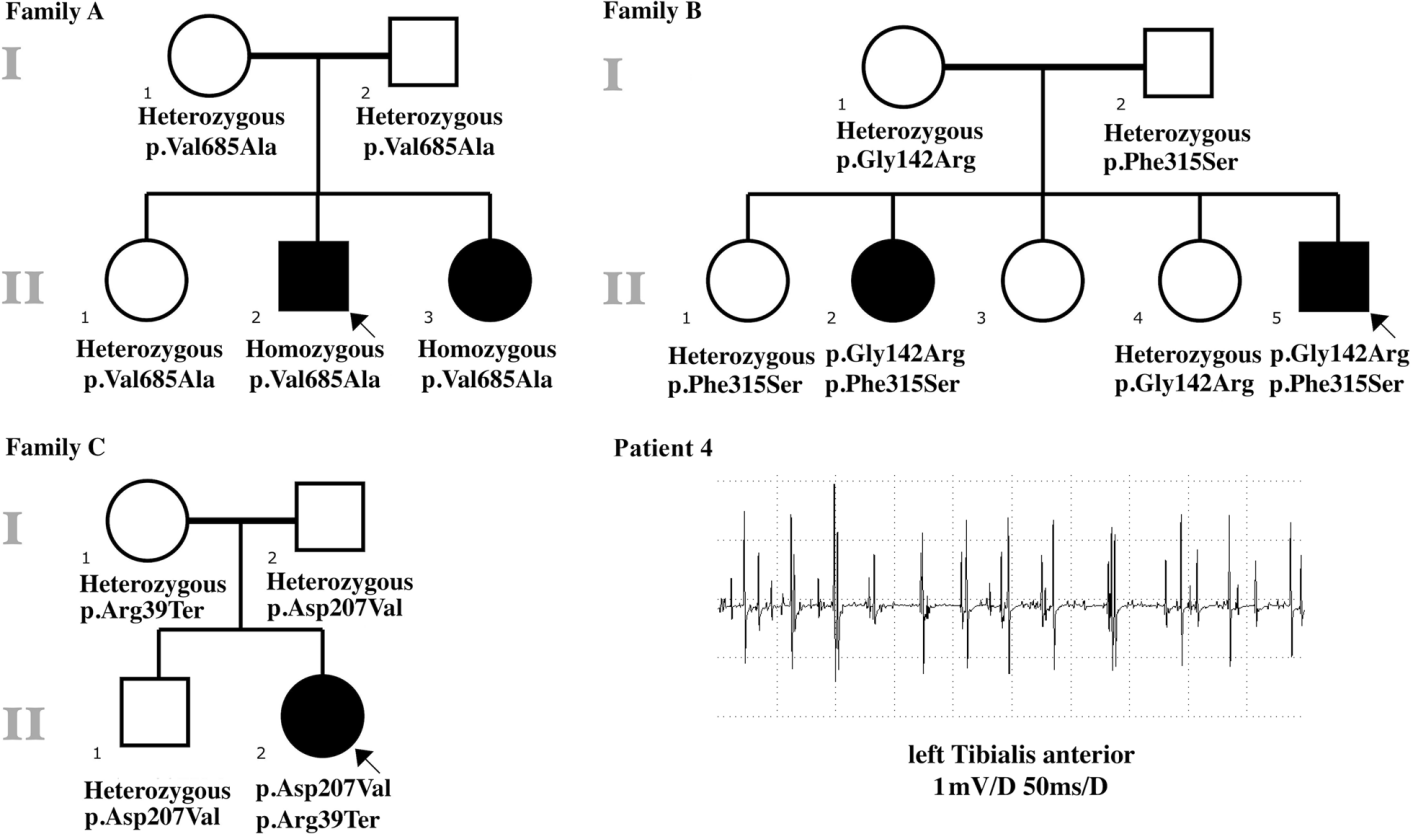
Pedigrees of families A-C and motor unit potentials of patient 4 in family C. The genotype was reported below available individuals. Reduced recruitment of the left tibialis anterior muscle of patient 4 was shown

A total of seven patients (patients 1 to 7) with GNE myopathy from six families were reviewed between April 2013 and August 2018. Neurogenic patterns of electrophysiology were identified in four patients (patients 1 to 4) from three families, and patients 5 to 7 were grouped into the control group. The clinical features and pedigrees for all patients are summarized in Table 1 and Fig. 1.

**Table 1:** Clinical characteristics and electrophysiology results of GNE myopathy patients with and without neurogenic patterns of electrophysiology

	Patient 1	Patient 2	Patient 3	Patient 4		Patient 5	Patient 6	Patient 7
*GNE* Mutation	p.Val685Ala, p.Val685Ala	p.Val685Ala, p.Val685Ala	p.Gly142Arg, p.Phe315Ser	p.Arg39*, p.Asp207Val		p.Arg39*, p.Asp207Val	p.Asp207Val, p.Val362Ala	p.Asp207Val, p.Lys240Glu
Age (y), gender	34, Male	31, Female	24, Male	25, Female		21, Female	26, Female	39, Female
Age at onset (y)	28	27	21	24		20	24	33
Lower limb reflex	Absent ankle reflex	Absent ankle reflex	NA	Absent patellar and ankle reflex		Absent patellar and ankle reflex	Absent patellar and ankle reflex	Absent patellar and ankle reflex
Creatine kinase	975 IU/L	NA	749 IU/L	276 IU/L		674 IU/L	351 IU/L	NA
Time between disease onset and EDX	6 years	5 years	3 years	1 year	1.3 years	1 year	3 years	6 years
Left tibial nerve CMAP amplitude (mV)	1.5↓	0.77↓	6.4	4.1	4.6	10.5	13.4	5.2
Right tibial nerve CMAP amplitude (mV)	1.1↓	0.32↓	5.4	6.9	7	10.5	19.7	4.5
Left peroneal nerve CMAP amplitude (mV)	0↓	0.20↓	0.3↓	0.72↓	0.58↓	3.2	5.8	2.4↓
Right peroneal nerve CMAP amplitude (mV)	0↓	0.19↓	0.7↓	5.1	2.7↓	4	7	3.3
EMG spontaneous activity (TA)	Fib 1 +	NA	Fib 3 +	NA	Fib 3 +	NA	Fib 0	Fib 3 +
	PSW 2 +		PSW 3 +		PSW 3 +		PSW 0	PSW 3 +
EMG Voluntary motor unit potentials (TA)	Amp↑(1059 μV)	NA	Amp↑	NA	Amp↑(1048 μV)	NA	NA	Amp (623 μV)
	Dur (11.6 ms)		Dur↑		Dur (12 ms)			Dur↓(6.1 ms)
	Recruitment↓↓		Recruitment↓↓		Recruitment↓↓↓			Complete recruitment with low amplitude

Neurogenic patterns were identified in patients 1 to 4. CMAP of the tibial nerves was recorded in the abductor hallucis muscle, and CMAP of the peroneal nerves was recorded in the extensor digitorum brevis muscle. Amp, amplitude of MUP. CMAP, compound motor action potential. Dur, Duration of MUP. EDX, Electrodiagnosis. EMG, Electromyography. Fib, fibrillations. MUP, motor unit potential. NA, not available. PSW, positive sharp waves. TA, tibialis anterior muscle

↓, mildly reduced; ↓↓, moderately reduced; ↓↓↓, severely reduced; ↑, increased;

Patient 1, the proband of family A (II:2, Fig. 1), was a 34-year-old male. Left big toe extensor weakness was first noticed at the age of 28 years. Paralysis progressed to the contralateral foot in two years and both hands in three years. On examination, pes cavus and severe atrophy of his tibialis anterior muscle were noticed. His strength was symmetrically reduced in the distal lower limbs with Medical Research Council (MRC) grade 3 in the calf and grade 1 in the tibialis anterior muscle (TA) and his ankle reflexes were absent on both sides without sensory disturbance. The patient had an elevated CK level of 975 IU/L. Patient 2 (II:3, Fig. 1) was the 3-year-younger sister of patient 1, who had similar symptoms in her 27 years. Her ankle reflexes were also absent on examination.

Patient 3 from family B was a 24-year-old male with weakness in his left foot for three years. Symptoms slowly developed in the right foot and both hands. He had pronounced weakness in his lower leg, with MRC grade 4 in the calf and grade 0 in the TA. CK level was 749 IU/L. One of his elderly sisters had similar weakness of the lower limb with a mildly elevated CK level of 204 IU/L. She did not visit our clinic, and the clinical phenotype and electrophysiology results were unavailable. We therefore excluded the patient from further analysis.

Patient 4 from family C was a 25-year-old female. She noticed left foot extensor weakness for one year and left ankle extensor weakness for seven months, while the strength of the upper limb was normal. She had atrophic left tibialis anterior muscle with reduced strength with MRC grade 4 and absent lower limb reflexes on both sides on examination. She had no family history, and her CK level was 276 IU/L.

Pictures of the lower limbs from patients 1, 4, 6, and 7 are shown in Additional file 1: Fig. 1. Images of lower limb MRI for patients 4 and 5 are shown in Additional file 1: Fig. 4. The lumbar MRI for patients 1 and 4 was normal. No history of disorders predisposing to neuropathy was identified, and extensive testing for acquired neuropathy was negative for all patients.

### Electrophysiology and pathology results

All patients completed at least one neurophysiology study (Table 1 and Additional file 1: Fig. 2). Patient 1 and his affected sister showed pronounced reduced CMAP amplitudes of the lower limb, and the peroneal nerve was more severe than the tibial nerve. Distal and proximal recordings of the left peroneal nerve were performed. The CMAP was 0 mV and 5.2 mV recording in the extensor digitorum brevis muscle and tibialis anterior muscle, respectively, showing length-dependent axonal neuropathy. The conduction velocity, upper limb, and sensory nerve studies were within the normal range. The EMG study of patient 1 showed moderate spontaneous potentials in the bilateral tibialis anterior muscle. The duration of motor unit potentials (MUPs) was normal, and the amplitude was increased in the tibialis anterior muscle, while the MUPs of the gastrocnemius muscle were normal. Patient 3 disclosed a reduced amplitude of CMAP of the peroneal nerve. The EMG of the bilateral tibialis anterior muscle revealed abundant spontaneous potentials and increased duration and amplitude of MUPs. The duration of gastrocnemius muscle MUP was increased. Patient 4 had decreased left peroneal CMAP in the first study, and the follow-up study revealed that the contralateral peroneal nerve was affected four months later. EMG of the gastrocnemius muscle and tibialis anterior muscle showed abundant spontaneous potentials. The amplitude of MUPs of the left tibialis anterior muscle was increased with reduced recruitment (Fig. 1) but was normal in the right tibialis anterior muscle and left gastrocnemius muscle. The clinical and neurophysiology profiles of the three patients without neurogenic patterns (patients 5 to 7) are also summarized in Table 1.

**Fig. 2:**
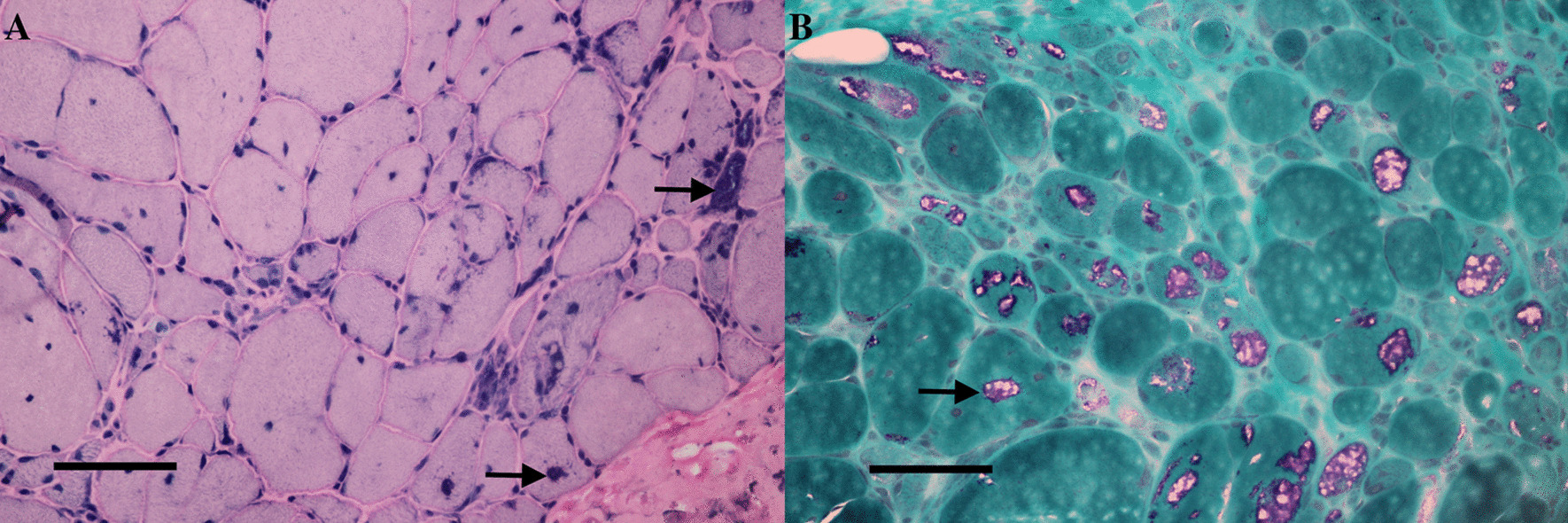
Left gastrocnemius muscle biopsy of patient 1 (II:2 in family A). (**A**) Hematoxylin and eosin staining revealed increased fiber size variability and small round and angular atrophy with vacuoles and basophilic material (arrows). (**B**) Modified Gomori trichrome staining showing rimmed cytoplasmic vacuoles (arrows). Scale bar = 100 μm in (**A**) and (**B**)

Patient 1 completed a left gastrocnemius muscle biopsy. Muscle H&E staining revealed a predominantly myogenic pattern, with small round and angular atrophic muscle fibers and atypical grouping. Rimmed vacuoles (RVs) were present in some of the atrophic fibers (Fig. 2). The expression of dystrophin, dysferlin, and sarcoglycans was normal. The H&E staining of sural nerve biopsy for patient 1 revealed no secondary peripheral neuropathy (Additional file 1: Fig. 3).

### Genetic analysis

Five missense variants of the *GNE* gene were identified in patients 1 to 4. Patient 1 and his affected sister carried homozygous c.2054T > C (p. Val685Ala) mutation, while their nonconsanguineous parents were both heterozygous. The c.2054T > C (p. Val685Ala) mutation located in the kinase coding region and was absent from the public population databases dbSNP, gnomAD, AbraOM, HEX, and GME. Prediction of the functional effect of the mutation suggests the deleterious effect of this variant (but is not necessarily pathogenic). The p.Val685Ala mutation was classified as likely pathogenic according to ACMG guidelines (for evidence PM2, PM3, PP1, and PP4). Patient 3 carried compound heterozygous variants of c.424G > A (p.Gly142Arg) and c.944T > C (p.Phe315Ser). Both variants were absent from population databases. All software predicted the p.Gly142Arg variant to be pathogenic and the p.Phe315Ser variant (rs1563938450) was predicted to be benign, and both variants were classified as uncertain significance according to ACMG guidelines (for evidence PM2, PP2 and PP4). Patient 4 carried compound heterozygous c.115C > T (p.Arg39*) and c.620A > T (p.Asp207Val) mutation. The c.115C > T(p.Arg39*) and c.620A > T(p.Asp207Val) mutations were previously reported as disease-causing mutations. Supported by PhyloP and PhastCons predictions, both p.Val685, p.Gly142 and p.Phe315 are highly conserved across species. The genetic results of the patients are summarized in Table 2.

**Table 2:** *GNE* mutations and functional effect predictions

Variants	Amino acid substituent	gnomAD frequency	SIFT prediction	Polyphen-2 prediction	MutationTaster prediction	CADD score	PhyloP	PhastCons
c.2054T > C	p.Val685Ala	0	Tolerated	Benign	Disease causing	23.4	4.117	1
c.424G > A	p.Gly142Arg	0	Deleterious	Probably damaging	Disease causing	26.8	5.161	1
c.944T > C	p.Phe315Ser	0	Tolerated	Benign	Disease causing	23.9	4.291	1
c.115C > T	p.Arg39*†	2/251278	Deleterious	Benign	Disease causing	37	4.014	1
c.620A > T	p.Asp207Val†	14/282714	Deleterious	Benign	Disease causing	23.8	2.781	1

^†^The two mutations were previously reported as disease-causing

## Discussion

In this study, we described rare neurogenic electrophysiology features in a series of patients with GNE myopathy. The absence of lower limb tendon reflexes, decreased CMAP amplitude, and neurogenic pattern of EMG distinguish these patients from typical myopathy.

The motor conduction study for patient 1 demonstrated severe axonal damage in the peroneal nerve in line with the tibialis anterior muscle predominant pattern of GNE myopathy. The electrophysiology feature was similar for his affected younger sister (patient 2), who carried the same mutation. Although the amplitude of CMAP of the tibial nerve of patient 3 was normal, EMG showed a neurogenic pattern in the gastrocnemius muscle, suggesting subclinical involvement of the tibial nerve. The p.Asp207Val mutation is a hotspot for the *GNE* gene in China, but axonal motor neuropathy was not observed in a cohort of 23 carriers [6]. Left peroneal nerve axonal neuropathy was observed in patient 4 at the first visit. However, a clear decrease in CMAP of the right peroneal nerve was noticed in the follow-up examination four months later, suggesting expanded degeneration of the distal motor nerve. There was no significant difference in clinical characteristics, such as onset age, sex, site of onset, disease duration, CK level, or EMG spontaneous potentials, between the patients with and without neurogenic features of electrophysiology. Features of axonal neuropathy, such as absent tendon reflexes, reduced CMAP of the peroneal nerve, occurrence of neurogenic and myopathic patterns in EMG, and denervation changes in muscle biopsies, have been reported in a number of cases and a recent case series of GNE myopathy [10–13]. We confirmed this feature in the present study. All four patients exhibited decreased CMAP of the lower limb, while the velocity, upper limb, and sensory were normal. We also found that the peroneal nerve was more vulnerable than the tibial nerve, and neurogenic MUPs were more common in the tibialis anterior muscle. Furthermore, follow-up results implied that CMAP decreased over time.

However, some neurogenic features of electrophysiology may also be present in myopathies. High-amplitude MUPs can be present among chronic myopathies, possibly due to the variation in fiber size [14]. Angulated atrophy may also be caused by chronic myopathies. In patient 1, although the electrophysiological manifestations were characteristic of a neurogenic pattern, muscle biopsy did not show typical fiber type grouping. This suggests that the neurogenic pattern may be caused by myopathy. Nevertheless, in the comparison of patients 4 and 5, although they had similar degrees of fat replacement and the same *GNE* gene mutations, there was a significant difference in the CMAP amplitude of the peroneal nerve between these two patients. This phenomenon also suggests that the decrease in CMAP amplitude may be caused by axonal neuropathy. However, due to the lack of muscle pathology, it is currently difficult to confirm whether these patients truly have neuropathy. Owing to the phenotypic variability of GNE myopathy, whether axonal motor neuropathy exists in these patients requires further follow-up. All four patients with neurogenic features were misdiagnosed with hereditary motor neuropathy in local clinics, despite having elevated CK levels. Therefore, we suggest routine screening for distal myopathies in patients with motor neuropathy and elevated CK levels.

We also discovered three novel variants in the *GNE* gene. The homozygous missense p.Val685Ala mutation was identified in two patients in family A. The pathogenicity of the p.Val685Ala mutation relies on the following evidence: (1) the mutation cosegregated with disease in this family; (2) the clinical features of distal myopathy, increased CK level, and RVs in atrophic fibers were consistent with GNE myopathy; and (3) the mutation was absent in the population database. The mutation was classified as likely pathogenic [15]. The p.Val685Ala variant is adjacent to the pathogenic mutation p.Ile687Asn and are both located in the ManNAc kinase domain. The compound heterozygous missense p.Gly142Arg and p.Phe315Ser variants of the *GNE* gene were found in patient 3 of family B. The novel compound heterozygous variants cosegregated with the distal myopathy phenotype in the family. Although the pattern of preferential tibialis anterior muscle involvement suggests the probability of GNE myopathy, we did not have pathology results to support the pathogenicity. Both variants were classified as Uncertain Significance. The p.Gly142Arg and p.Phe315Ser variants are both located in the UDP-GlcNAc 2-epimerase domain of the GNE protein near the pathogenic mutation p.Ile137Thr and p.Pro314Ser. All three novel variants were evolutionarily conserved, suggesting the pathogenicity of these variants. Further work is still required to establish the relationship between the disease phenotype and these novel variants.

The mechanism responsible for this rare electrophysiological manifestation is still debated. GNE is the rate-limiting enzyme of sialic acid biosynthesis. Although heterozygously mutated GNE reduces the sialylation level in a mouse model of the disease, the phenotype is normal [16]. The hyposialylation of specific glycoproteins and glycolipids in muscle may contribute to the pathophysiology of GNE myopathy. Decreased sialylation has also been reported in alpha-dystroglycan [17] and neural cell adhesion molecules [18]. Sialic acid also has a close relationship with ganglioside GM3 [19], which plays a key role in neurons. It is possible, therefore, that hyposialylation of gangliosides in motor nerve axons may cause axonal neuropathy, which could be a possible mechanism for the neurogenic features of electrophysiology.

## Conclusions

In summary, we described a series of patients with GNE myopathy accompanied by neurogenic features of electrophysiology. The pathogenicity of p.Gly142Arg, p.Phe315Ser, and p.Val685Ala mutation in the *GNE* gene needs confirmation. The pathophysiology of this phenomenon still warrants further investigation.

## Methods

### Patients

We retrospectively collected gene-diagnosed patients with GNE myopathy and their affected family members from 2013 to 2018 in the neuromuscular clinic of Peking University Third Hospital. All patients’ medical histories, including onset age, presenting symptoms, and neurological examination of motor and sensory function, as well as laboratory studies of serum creatine kinase (CK) levels and tests for secondary peripheral neuropathy, were reviewed. A detailed family history was also reviewed.

### Electrophysiology and pathology evaluation

Electrophysiology was performed in all patients, including a standard motor and sensory conduction velocity (MCV and SCV), compound motor action potential (CMAP) amplitude, and H-reflex of the bilateral tibial nerve. Electromyography (EMG) of the tibialis anterior muscle, gastrocnemius muscle and upper limb muscles was evaluated. The neurogenic pattern of electrophysiology was defined when the MCV/SCV or CMAP amplitude was reduced below the lower limit of normal value, with high-amplitude MUP or reduced recruitment. The age-matched normal values of the peroneal nerves and tibial nerves are listed in the Additional file 1. Gastrocnemius muscle and sural nerve biopsies were available for one patient with a neurogenic pattern of electrophysiology. Standard staining for hematoxylin and eosin (H&E), Gomori trichrome, ATPase, Oil Red O, nicotinamide adenine dinucleotide-tetrazolium reductase (NADH-TR), and Periodic acid Schiff (PAS) was performed. Additionally, immunohistochemistry was routinely performed to identify dystrophin, dysferlin, and sarcoglycans to rule out muscular dystrophies.

### Gene sequencing and bioinformatic analysis

Genomic DNA samples from all subjects and family members were extracted from peripheral blood leukocytes using a DNA Isolation Kit (Bioteke, AU1802). Whole-exome sequencing or a panel for 199 genes of hereditary myopathies and 159 genes of peripheral neuropathies (Additional file 1: Table 1) was sequenced using exome capture sequencing technology on HiSeq X10 (Illumina, San Diego, USA) sequencers (sequenced in Beijing Running Gene Inc., the variant filtering criteria for WES data are listed in the Additional file 1). The detected *GNE* gene variants were validated by polymerase chain reaction.

The variants and amino acid substituents are described in the current nomenclature (GenBank NM_001128227 and NP_001121699). The minor allele frequency of variants was identified in the population database of Short Genetic Variations (dbSNP http://www.ncbi.nlm.nih.gov/SNP/), the Genome Aggregation Database (gnomAD, http://gnomad.broadinstitute.org), the Arquivo Brasileiro Online de Mutações (AbraOM, http://abraom.ib.usp.br), the Healthy Exomes database (HEX, http://www.alzforum.org/exomes/hex), and the Greater Middle East Variome Project (GME, http://gme.igm.ucsd.edu). The Human Gene Mutation Database was used to determine the novelty of variants. The MutationTaster (http://www.mutationtaster.org), PolyPhen-2 (http://genetics.bwh.harvard.edu/pph2), SIFT (http://sift.jcvi.org/), and CADD (http://cadd.gs.washington.edu) programs were used to evaluate deleterious effects of the novel *GNE* gene variants. We measured evolutionary conservation using PhyloP and PhastCons (http://compgen.bscb.cornell.edu/phast/) software. The pathogenicity of the novel variants was interpreted according to the American College of Medical Genetics and Genomics (ACMG) guidelines [15].

## Supplementary Information

**Additional file 1: 1. Supplementary Table.** List of gene panels for peripheral neuropathies (159 genes) and hereditary myopathies (199 genes).** 2.** The variant annotation and filtering criteria of whole exome sequencing.** 3.** The motor nerve conduction studies of the lower limb.** 4.** The age-matched normal value of the laboratory.** 5. Supplementary figure 1**. The lower limbs of patients with GNE myopathy.** 6. Supplementary figure 2**. The waveforms of motor nerve conduction studies of the lower limb.** 7. Supplementary figure 3**. H&E staining of sural nerve biopsy for patient 1 revealed no secondary peripheral neuropathy. **8.Supplementary figure 4**. Images of lower limb MRI for patient 4 and 5.

## Data Availability

Data will be available upon request.

## References

[1] Nishino I, Noguchi S, Murayama K, Driss A, Sugie K, Oya Y (2002). Distal myopathy with rimmed vacuoles is allelic to hereditary inclusion body myopathy. Neurology.

[2] Eisenberg I, Avidan N, Potikha T, Hochner H, Chen M, Olender T (2001). The UDP-N-acetylglucosamine 2-epimerase/N-acetylmannosamine kinase gene is mutated in recessive hereditary inclusion body myopathy. Nat Genet.

[3] Hinderlich S, Stasche R, Zeitler R, Reutter W (1997). A bifunctional enzyme catalyzes the first two steps in N-acetylneuraminic acid biosynthesis of rat liver. Purification and characterization of UDP-N-acetylglucosamine 2-epimerase/N-acetylmannosamine kinase. J Biol Chem..

[4] Celeste FV, Vilboux T, Ciccone C, de Dios JK, Malicdan MC, Leoyklang P (2014). Mutation update for GNE gene variants associated with GNE myopathy. Hum Mutat.

[5] Argov Z, Eisenberg I, Grabov-Nardini G, Sadeh M, Wirguin I, Soffer D (2003). Hereditary inclusion body myopathy: the Middle Eastern genetic cluster. Neurology.

[6] Chen Y, Xi J, Zhu W, Lin J, Luo S, Yue D (2019). GNE myopathy in Chinese population: hotspot and novel mutations. J Hum Genet.

[7] Reinke SO, Lehmer G, Hinderlich S, Reutter W (2009). Regulation and pathophysiological implications of UDP-GlcNAc 2-epimerase/ManNAc kinase (GNE) as the key enzyme of sialic acid biosynthesis. Biol Chem.

[8] Izumi R, Niihori T, Suzuki N, Sasahara Y, Rikiishi T, Nishiyama A (2014). GNE myopathy associated with congenital thrombocytopenia: a report of two siblings. Neuromuscul Disord.

[9] Chai Y, Bertorini TE, McGrew FA (2011). Hereditary inclusion-body myopathy associated with cardiomyopathy: report of two siblings. Muscle Nerve.

[10] Huang YN, Chuang HJ, Hsueh HW, Huang HC, Lee NC, Chao CC (2020). A case of GNE myopathy mimicking hereditary motor neuropathy. Eur J Neurol.

[11] Grecu N, Villa L, Cavalli M, Ristaino A, Choumert A, Butori C (2021). Motor axonal neuropathy associated with GNE mutations. Muscle Nerve.

[12] Previtali SC, Zhao E, Lazarevic D, Pipitone GB, Fabrizi GM, Manganelli F (2019). Expanding the spectrum of genes responsible for hereditary motor neuropathies. J Neurol Neurosurg Psychiatry.

[13] Zhao J, Wang Z, Hong D, Lv H, Zhang W, Chen J (2015). Mutational spectrum and clinical features in 35 unrelated mainland Chinese patients with GNE myopathy. J Neurol Sci.

[14] Rowinska-Marcinska K, Szmidt-Salkowska E, Fidzianska A, Zalewska E, Dorobek M, Karwanska A (2005). Atypical motor unit potentials in Emery-Dreifuss muscular dystrophy (EDMD). Clin Neurophysiol.

[15] Richards S, Aziz N, Bale S, Bick D, Das S, Gastier-Foster J (2015). Standards and guidelines for the interpretation of sequence variants: a joint consensus recommendation of the American college of medical genetics and genomics and the association for molecular pathology. Genet Med.

[16] Gagiannis D, Orthmann A, Danssmann I, Schwarzkopf M, Weidemann W, Horstkorte R (2007). Reduced sialylation status in UDP-N-acetylglucosamine-2-epimerase/N-acetylmannosamine kinase (GNE)-deficient mice. Glycoconj J.

[17] Huizing M, Rakocevic G, Sparks SE, Mamali I, Shatunov A, Goldfarb L (2004). Hypoglycosylation of alpha-dystroglycan in patients with hereditary IBM due to GNE mutations. Mol Genet Metab.

[18] Ricci E, Broccolini A, Gidaro T, Morosetti R, Gliubizzi C, Frusciante R (2006). NCAM is hyposialylated in hereditary inclusion body myopathy due to GNE mutations. Neurology.

[19] Paccalet T, Coulombe Z, Tremblay JP (2010). Ganglioside GM3 levels are altered in a mouse model of HIBM: GM3 as a cellular marker of the disease. PLoS ONE.

