# Supplementary material for: Different electrophysiology patterns in GNE myopathy
Source: Orphanet J Rare Dis. 2022 May 19;17:206. doi: 10.1186/s13023-022-02355-0 (PMC9118620; doi:10.1186/s13023-022-02355-0)
Supplement: Supplementary file 1 — Additional file 1: 1. Supplementary Table. List of gene panels for peripheral neuropathies (159 genes) and hereditary myopathies (199 genes). 2. The variant annotation and filtering criteria of whole exome sequencing. 3. The motor nerve conduction studies of the lower limb. 4. The age-matched normal value of the laboratory. 5. Supplementary figure 1. The lower limbs of patients with GNE myopathy. 6. Supplementary figure 2. The waveforms of motor nerve conduction studies of the lower limb. 7. Supplementary figure 3. H&E staining of sural nerve biopsy for patient 1 revealed no secondary peripheral neuropathy. 8.Supplementary figure 4. Images of lower limb MRI for patient 4 and 5. [file 13023_2022_2355_MOESM1_ESM.docx]

**Different electrophysiology patterns in GNE myopathy**

**Supplementary Material**

1. **Supplementary Table. List of gene panels for peripheral neuropathies (159 genes) and hereditary myopathies (199 genes).**

Peripheral neuropathies (159 genes):

| AAAS | AARS | ABCA1 | ABCD1 | ABHD12 | ACO2 | AGL |
| --- | --- | --- | --- | --- | --- | --- |
| AIFM1 | AMACR | ARHGEF10 | ARSA | ASAH1 | ATL1 | ATL3 |
| ATP1A1 | BAG3 | BICD2 | BSCL2 | CAV1 | CCT5 | CD59 |
| CLCN7 | COX6A1 | CTDP1 | CUL4B | CYP27A1 | DCAF8 | DGAT2 |
| DHH | DHTKD1 | DNAJB2 | DNAJC3 | DNM2 | DNMT1 | DRP2 |
| DST | DYNC1H1 | EGR2 | ERCC6 | ERCC8 | EXOSC3 | FAH |
| FAM126A | FBLN5 | FGD4 | FIG4 | FLVCR1 | GALC | GAN |
| GARS | GBE1 | GDAP1 | GJB1 | GJB3 | GLA | GNB4 |
| GRN | HARS | HEXA | HINT1 | HK1 | HNRNPA2B1 | HOXD10 |
| HSD17B4 | HSPB1 | HSPB3 | HSPB8 | IFRD1 | IGHMBP2 | IKBKAP |
| INF2 | KARS | KIF1A | KIF1B | KIF5A | KLHL9 | LAS1L |
| LITAF | LMNA | LRSAM1 | MARS | MCM3AP | MED25 | MFN2 |
| MMACHC | MME | MORC2 | MPV17 | MPZ | MTMR2 | MTTP |
| MYH14 | NAGLU | NALCN | NDRG1 | NEFH | NEFL | NGF |
| NGLY1 | NPC1 | NPC2 | NTRK1 | PDK3 | PEX1 | PEX7 |
| PHYH | PLA2G6 | PLEKHG5 | PMM2 | PMP2 | PMP22 | PRDM12 |
| PRNP | PRPS1 | PRX | PTRH2 | RAB7A | RBM28 | REEP1 |
| RETREG1 | RNF170 | RPIA | SAR1B | SBF1 | SBF2 | SCN11A |
| SCN9A | SCO2 | SCP2 | SCYL1 | SEPT9 | SGPL1 | SH3TC2 |
| SIL1 | SLC12A6 | SLC25A46 | SMPD1 | SNAP29 | SOX10 | SPG11 |
| SPTLC1 | SPTLC2 | SURF1 | TDP1 | TFG | TMEM126A | TRIM2 |
| TRPV4 | TUBB3 | TWNK | VCP | VRK1 | WARS | WNK1 |
| YARS | FRDA | DSTYK | LGI4 | SETX |  |  |

Hereditary myopathies (199 genes):

| ACTA1 | ACTG2 | ACVR1 | ADCY5 | ADCY6 | ADGRG6 | AGRN |
| --- | --- | --- | --- | --- | --- | --- |
| ALG14 | ALG2 | AMPD1 | ANO5 | ATP2A1 | B3GALNT2 | B4GAT1 |
| BAG3 | BIN1 | BVES | CACNA1A | CACNA1S | CAPN3 | CASQ1 |
| CASQ2 | CAV3 | CCDC78 | CFL2 | CHAT | CHCHD10 | CHKB |
| CHRNA1 | CHRNB1 | CHRND | CHRNE | CLCN1 | INPP5K | CNTN1 |
| CNTNAP1 | COA6 | COL12A1 | COL13A1 | COL25A1 | COL6A1 | COL6A2 |
| COL6A3 | COLQ | CPT2 | CRYAB | DAG1 | DES | DMD |
| ZBTB42 | DNA2 | DNAJB6 | DNM2 | DOK7 | DPAGT1 | DYSF |
| ECEL1 | EMD | ERBB3 | FAM111B | FBN2 | FHL1 | FKBP14 |
| FKRP | FKTN | FLAD1 | FLNC | GAMT | GFER | GFPT1 |
| GLDN | GLE1 | GMPPB | GNE | GYG1 | HACD1 | HINT1 |
| HNRNPA2B1 | HNRNPDL | HRAS | ISCU | ISPD | ITGA7 | KBTBD13 |
| KCNA1 | KCNA2 | KCNE1 | KCNE3 | KCNH2 | KCNJ16 | KCNJ18 |
| KCNJ2 | KCNJ5 | KCNQ1 | KCNQ2 | KIF21A | KLHL40 | KLHL41 |
| KY | LAMA2 | LARGE1 | LDB3 | LIMS2 | LMNA | LMOD3 |
| LPIN1 | LRP4 | MEGF10 | MICU1 | MSTN | MTM1 | MUSK |
| MYBPC1 | MYF6 | MYH14 | MYH2 | MYH3 | MYH7 | MYH8 |
| MYO18B | MYOT | MYPN | NALCN | NEB | NEK9 | NOL3 |
| NOTCH3 | OPA1 | ORAI1 | PABPN1 | PDGFRB | PHOX2A | PIEZO2 |
| PIP5K1C | PLEC | PNPLA2 | PNPLA8 | POGLUT1 | POLG | POLG2 |
| POMGNT1 | POMGNT2 | POMK | POMT1 | POMT2 | PREPL | PUS1 |
| PYROXD1 | RAPSN | RBCK1 | RNASEH1 | RRM2B | RYR1 | RYR2 |
| SCN1A | SCN4A | SCN5A | SELENON | SGCA | SGCB | SGCD |
| SGCE | SGCG | SLC18A3 | SLC25A4 | SLC5A7 | SLC6A8 | SMCHD1 |
| SNAP25 | SPEG | STAC3 | STIM1 | SUCLA2 | SYNE1 | SYNE2 |
| SYT2 | TBCE | TCAP | TIA1 | TMEM43 | TMEM5 | TNNI2 |
| TNNT1 | TNNT3 | TNPO3 | TOR1AIP1 | TPM2 | TPM3 | TRAPPC11 |
| TRDN | TRIM32 | TRIP4 | TTN | TUBB3 | TWNK | VIPAS39 |
| VMA21 | VPS33B | YARS2 |  |  |  |  |

1. **The variant annotation and filtering criteria of whole exome sequencing:**

Quality control was applied to raw data (stored in FASTQ format) obtained from HiSeq X10 to guarantee the meaningfulness of downstream analysis. The steps of data processing were as follows: (i) filter reads with adapter contamination (>10 nucleotides aligned to the adapter, allowing ≤ 10% mismatches); (ii) discard reads containing ≥ 10% unidentified nucleotides; and (iii) discard the paired reads when a single read has more than 50% low quality (Phred quality <5) nucleotides. The percentage of reads with average quality >Q30 and GC content distribution were calculated and summarized. High-quality paired-end reads were aligned to the human reference genome sequence from the UCSC database (build 37.1 version hg19, http://genome.ucsc.edu/) using the Burrows-Wheeler Alignment tool. We estimated quality scores and made consensus SNPs and insertions and deletions (indels) calls using GATK. Low-quality variations were filtered out using the following criteria: (i) QD (quality by depth) <2.0; (ii) MQ (mapping quality) <40.0; (iii) FS (Fisher strand) >60.0; (iv) MQRankSum (mapping quality rank sum test) <-12.5; and (v) ReadPosRankSum (read position rank sum test) <-8.0. All the called variants were annotated using several public databases (1000 Genomes Project, ExAC, gnomAD, ESP6500, CCDS, RefSeq, Ensembl, etc.). Annotation content contained the variant position, variant type, allele frequency, conservative prediction, etc., which would help to locate mutations relative to diseases. The obtained variants were further selected according to cosegregation, genetic modeling and an MAF <1% in three databases (1000 Genomes Project_EAS, ExAC, gnomAD_EAS). SNPs and indels occurring in exons and canonical splice sites were further analyzed.

1. **The motor nerve conduction studies of the lower limb.**

The room temperature was >28℃, and the foot temperature was >31℃.

- Peroneal nerve conduction study (motor)

1. A pair of recording electrodes are used with the active lead placed on the belly of the extensor digitorum brevis (EDB) muscle and the reference lead on the tendon;
2. The nerve was stimulated with supramaximal stimulation;
3. Stimulate the deep peroneal nerve at the ankle;
4. Stimulate the common peroneal nerve at the knee;
5. If the action potential was not recorded in EDB, then the recording site was placed on the tibialis anterior muscle, and the nerve was stimulated at the knee.

- Tibial nerve conduction study (motor)

1. A pair of recording electrodes are used with the active lead placed on the belly of the abductor hallucis muscle and the reference lead on the tendon;
2. The nerve was stimulated with supramaximal stimulation;
3. Stimulate the deep peroneal nerve at the ankle;
4. Stimulate the common peroneal nerve at the knee.
5. If the action potential was not recorded in AH, then place the recording site on the calf muscle and stimulate the nerve at the knee.
6. **The age-matched normal value of the laboratory.**

The lower limit of normal value of motor peroneal nerves

| Stimulation site | Age | Latency(ms) | Amplitude(mV) | Velocity(m/s) |
| --- | --- | --- | --- | --- |
| Ankle | 15-65 | 3.4 | 3 |  |
| Knee | 15-45 |  |  | 44 |
| Knee(record at TA) | 15-65 |  | 6 |  |

The lower limit of normal value of motor tibial nerves

| Stimulation site | Age | Latency(ms) | Amplitude(mV) | Velocity(m/s) |
| --- | --- | --- | --- | --- |
| Ankle | 15-25 | 3.0 | 6 |  |
|  | 40-65 | 2.7 | 4 |  |
| Knee | 15-25 |  |  | 45 |
|  | 40-65 |  |  | 43 |
| Knee(record at calf) | 15-25 | 4.3 | 6 |  |
|  | 40-55 | 3.9 | 7 |  |

1. **Supplementary figure 1. The lower limbs of patients with GNE myopathy.**


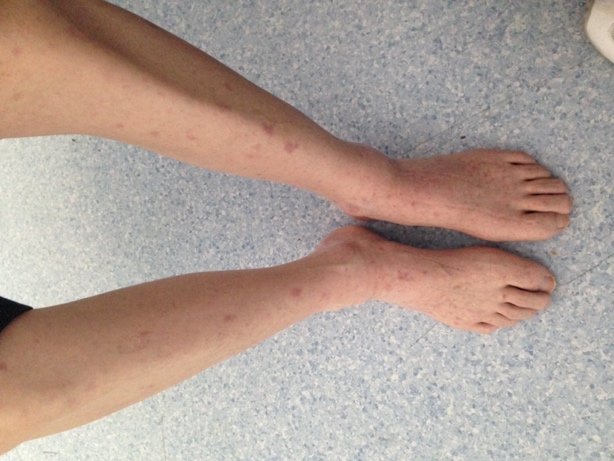


Patient 1


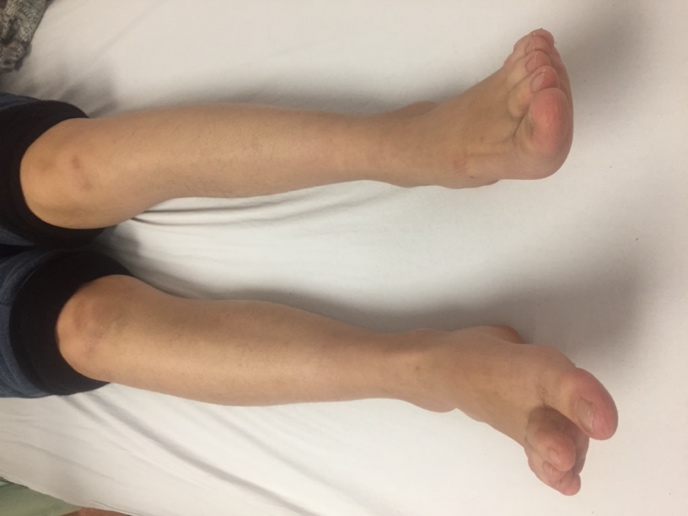


Patient 4


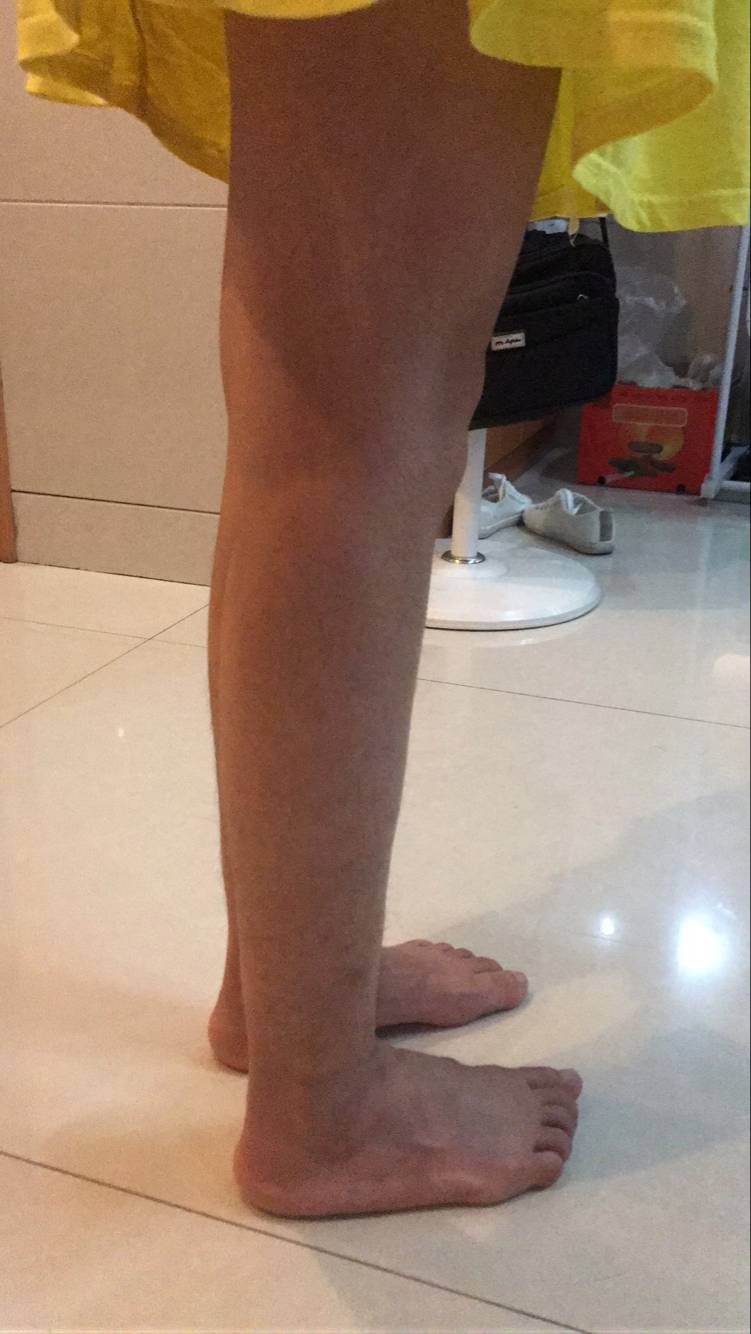


Patient 6


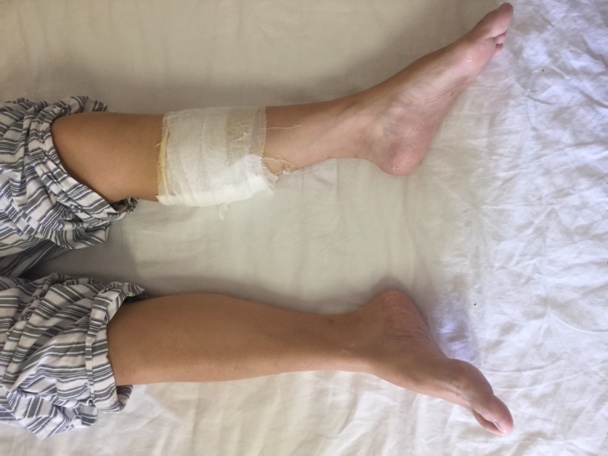


Patient 7

1. **Supplementary figure 2. The waveforms of motor nerve conduction studies of the lower limb.**


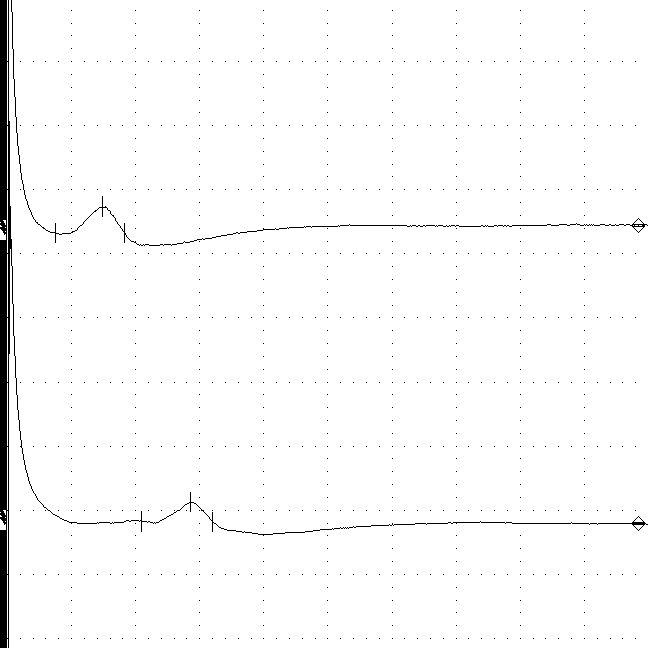


Patient 2, Left peroneal nerve motor conduction study, 0.5mV/D.

Upper curve: action potential evoked when stimulating the deep peroneal nerve at the ankle.

Lower curve: action potential evoked when stimulating the common peroneal at the knee.


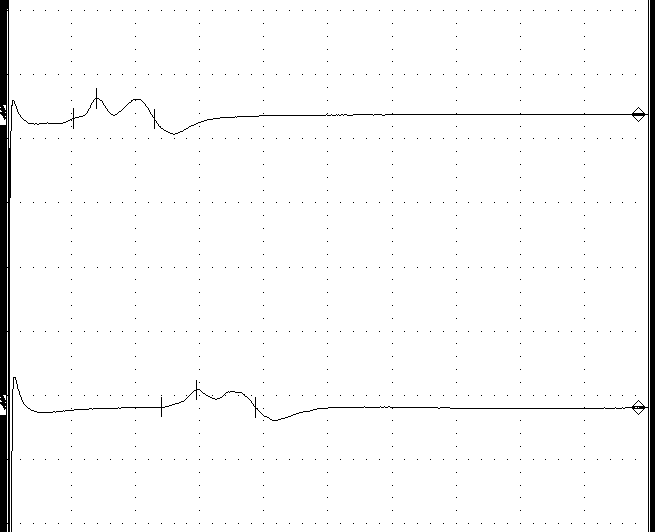


Patient 2, Right tibial nerve motor conduction study, 1mV/D.

Upper curve: action potential evoked when stimulating the tibial nerve at the ankle.

Lower curve: action potential evoked when stimulating the tibial nerve at the knee.


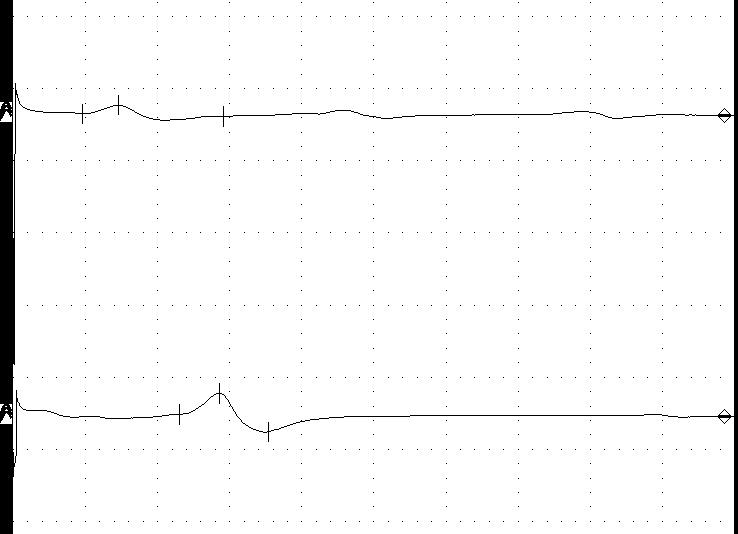


Patient 4, Left peroneal nerve motor conduction study, 5mV/D.

Upper curve: action potential evoked when stimulating the deep peroneal nerve at the ankle.

Lower curve: action potential evoked when stimulating the common peroneal at the knee.

1. **Supplementary figure 3. H&E staining of sural nerve biopsy for patient 1 revealed no secondary peripheral neuropathy.**


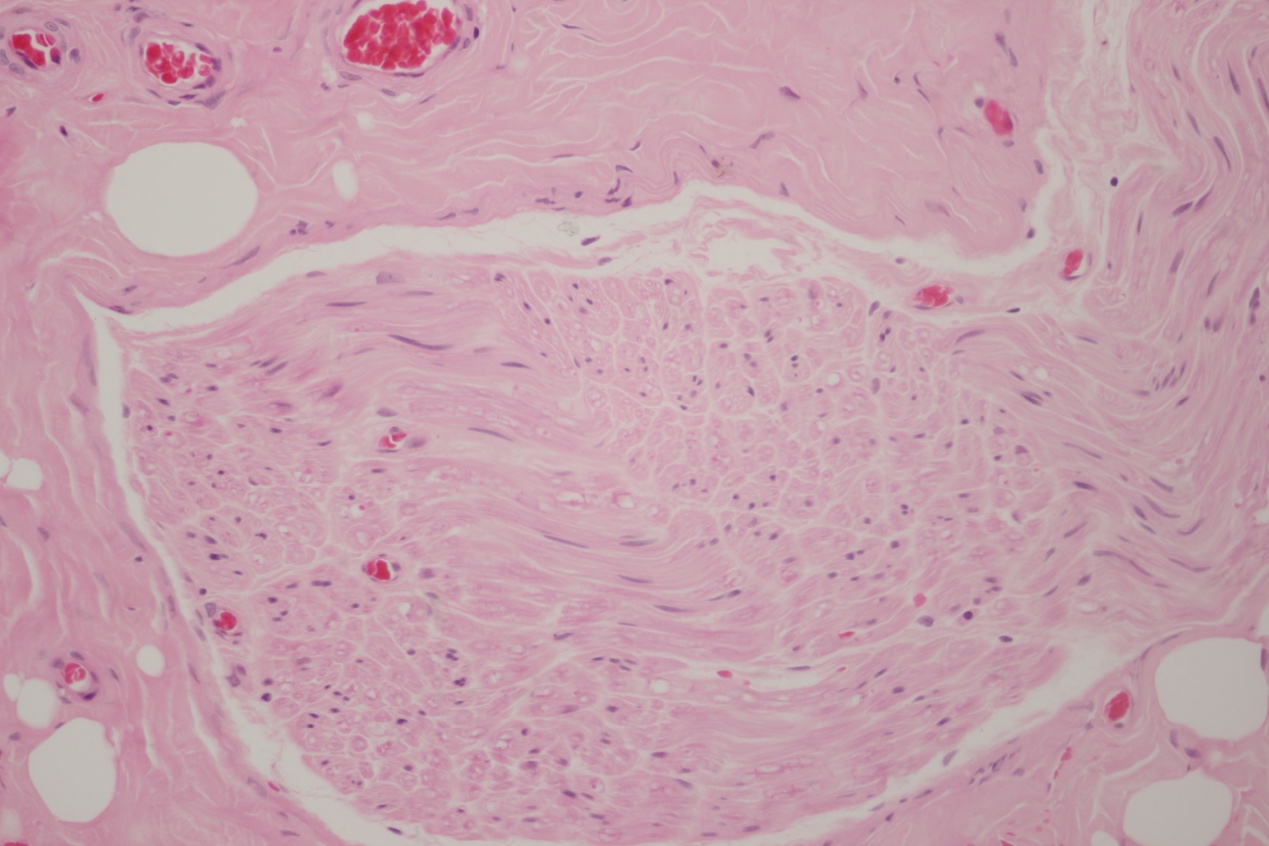


1. **Supplementary figure 4. Images of lower limb MRI for patient 4 and 5.**


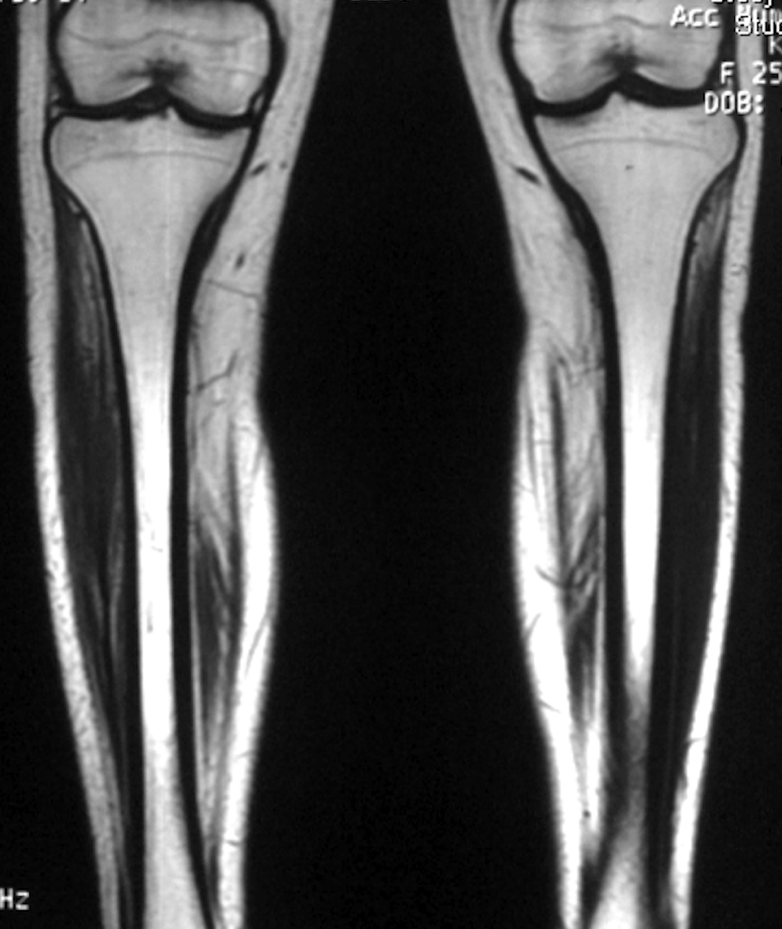


Patient 4, T1WI of Lower leg.


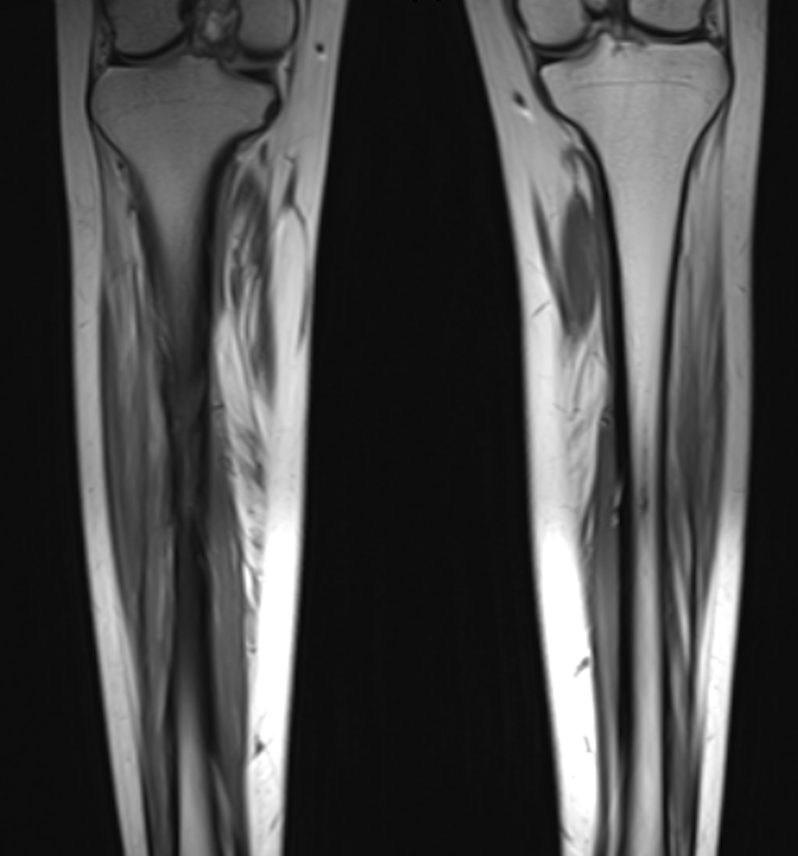


Patient 5, T1WI of Lower leg.
